# Innovative vision rehabilitation method for hemianopsia: Comparing pre- and post audio-luminous biofeedback training for ocular motility improving visual functions and quality of life

**DOI:** 10.3389/fneur.2023.1151736

**Published:** 2023-04-11

**Authors:** Mariana Misawa, Yulia Pyatova, Atri Sen, Michelle Markowitz, Samuel N. Markowitz, Michael Reber, Monica Daibert-Nido

**Affiliations:** ^1^Toronto Western Hospital, University Health Network, Toronto, ON, Canada; ^2^Department of Ophthalmology and Vision Science, Faculty of Medicine, University of Toronto, Toronto, ON, Canada; ^3^Department of Occupational Science and Occupational Therapy, University of Toronto, Toronto, ON, Canada; ^4^Donald K. Johnson Eye Institute, Krembil Research Institute, University Health Network, Toronto, ON, Canada

**Keywords:** biofeedback training - BT, hemianopia or hemianopsia, rehabilitation, vision, visual fields, microperimetry sensitivity, fixation stability, quality of life

## Abstract

**Background:**

Homonymous hemianopsia (HH) corresponds to vision loss in one hemi-field secondary to retro-chiasmal injury. Patients with HH experience difficulties in scanning and orientation in their environment. Near vision daily activities such as reading can also be impaired. There is an unmet need for standardized vision rehabilitation protocols for HH. We investigated the effectiveness of biofeedback training (BT), used for vision rehabilitation in patients with central vision loss, in individuals with HH.

**Methods:**

In this prospective pilot pre/post study, 12 participants, with HH consecutive to brain injury, performed 5 weekly BT sessions for 20 min each under supervision using the Macular Integrity Assessment microperimeter. BT consisted of relocation of the retinal locus 1–4° toward the blind hemi-field. Outcomes measured post-BT were paracentral retinal sensitivity, visual acuity (near vision), fixation stability, contrast sensitivity, reading speed, and visual functioning questionnaire. Statistical analysis was performed using Bayesian paired t-tests.

**Results:**

Paracentral retinal sensitivity significantly increased by 2.7 ± 0.9 dB in the treated eye in 9/11 of the participants. Significant improvements with medium-to-large effect size were observed for fixation stability (8/12 participants), contrast sensitivity (6/12 participants) and near vision visual acuity (10/12 participants). Reading speed increased by 32.5 ± 32.4 words per minute in 10/11 participants. Quality of vision scores improved significantly with large effect size for visual ability, visual information and mobility.

**Conclusion:**

BT led to encouraging improvements in visual functions and functional vision in individuals with HH. Further confirmation with larger trials is required.

## Introduction

Patients with brain injury frequently suffer from homonymous hemianopsia (HH), defined as vision loss in one vertical hemi-field secondary to a retro-chiasmal lesion (1–4). In HH, impaired eye movements lead also to defective visual and spatial scanning and exploration. This defective scanning on the blind hemifield affects orientation and mobility, the ability to walk independently and as such, the quality of life (1–3). Moreover, because of the visual field loss, the subjective midline deviates affecting balance and contributing to an increase in risk of falling (5).

Central vision can also be affected in hemianopsia due to a parafoveal field loss with splitting of fixation and poor eye movements leading to reading deficit. Left-to-right readers with a right HH have particularly impaired reading abilities. For efficient reading, three to four letters to the left and seven to 11 letters to the right of fixation must be seen (1, 4, 6). Patients with right HH have trouble locating ensuing words, making systematic saccades to find those words. Additionally, there is prolonged fixation, disrupted saccadic amplitude, and an increased number of regressive saccades. Because parafoveal vision is used to obtain information about forthcoming words, patients with 3°–5° of macular sparing tend to have minimal impairment of reading (1, 4, 6).

Patients with HH naturally develop oculomotor strategies to compensate for visual field loss, however, these strategies are often suboptimal and oculomotor control is impaired (1). To scan the blind hemi-field, patients perform dismetric saccades with increased amplitude (7, 8). Fixation stability and landing accuracy decrease, affecting visual acuity (9, 10).

Biofeedback training (BT) is a compensatory rehabilitation technique that emerged three decades ago and has been used in various fields of medicine, including ophthalmology (11, 12). BT is one of the newest and more modern low vision rehabilitation techniques. By increasing the oculomotor control and relocating the visual fields through a change in the patient’s locus of fixation, BT improves the visual acuity for distance, near vision, contrast sensitivity, retinal sensitivity, reading speed, and quality of life in many eye conditions. Mounting evidence has highlighted the benefits from BT in age-related macular degeneration, myopic degeneration, Stargardt’s disease, glaucoma, and nystagmus (13–19). The studies show similar effectiveness using 4–10 sessions of BT, varying from 10 to 20 min each, although a minority of them followed the patients on a long-term basis (15). Our department has treated more than 350 individuals with low vision using BT with benefits sustained up to 5 years. However, BT had never been used for visual rehabilitation in hemianopsia before this study.

The goal of this study was to use BT in an innovative way. Using our experience from different pathologies, we proposed a BT protocol specific to HH with the automated MAIA microperimeter. One of the symptoms in hemianopia is the loss of parafoveal visual references, causing oculomotor dysfunction. We hypothesized that relocating the fixation locus of the patients to an area with a larger span would improve oculomotor control and increase visual performance. A retinal locus with higher sensitivity and larger visual span was used on the seeing hemi-retina, or blind hemifield. A maximum of 4° of fixation relocation was allowed, in order to keep the better visual acuity from the parafoveal retina. This method would eliminate the splitting of fixation from the hemianopsia while improving the oculomotor function. BT is a technique that primarily increases the fixation stability and oculomotor control, perpetuating the benefit from field relocation.

## Materials and methods

### Study design/participants

The study was designed as a prospective, pilot, interventional pre/post, case series. Twelve patients were recruited from the Low Vision Clinic at the Toronto Western Hospital, University of Toronto, Canada. Criteria for inclusion were diagnosis of hemianopsia based on visual fields, brain injury from various etiologies, age between 18 and 90 years old, and ability to follow the visual, auditory stimuli, and training instructions. Exclusion criteria were previous treatment for low vision rehabilitation, significant underlying ocular pathology not related to the hemianopsia physiopathology, and cognitive impairment that prevents an adequate test and training performance. Patients had one baseline visit 1 (V1), five BT visits (V2-6), 1 week follow up visit (V7) and 1 month follow up visit (V8).

Twelve patients (Table 1) with hemianopsia were treated with 5 BT sessions (100 min in total) over a 5-week period without adverse events. Age ranged from 40 to 90 years old (average 66.6 ± 15.3). 58% of the subjects were female. The time post-brain injury ranged from 2.5 to 36 months, average 12.3 ± 9.5 months. Only P5 had less than 5 months from the injury. Most of the patients (9) had a stroke. None of the patients had macular sparing as defined by a 4° parafoveal normal sensitivity. Eight patients had a left hemianopsia, three had a right hemianopsia, and P10 had a bitemporal hemianopsia. The eye ipsilateral to the blind hemi-field and, for P10, the eye with the best fixation stability, was treated. P7 was excluded for being unable to comply with the tests (Table 1) and paracentral retinal sensitivity could not be recorded in P8 due to dizziness.

**Table 1:** Participants’ demographic.

ID	Age range	Sex	Ethnicity	Cause	Side	Time from event
P1	50s	M	Black	Stroke	L	7 months
P2	40s	F	Caucasian	Stroke	L	12 months
P3	70s	M	Caucasian	Herpetic Encephalitis	L	7 months
P4	60s	F	Caucasian	Neurosurgery	R	5 months
P5	80s	M	Latino	Stroke	L	2.5 months
P6	90s	F	Caucasian	Stroke	R	24 months
P8	50s	F	Caucasian	Stroke	L	10 months
P9	80s	F	Caucasian	Stroke	L	36 months
P10	60s	M	Asian	Neurosurgery	B	18 months
P11	80s	F	Caucasian	Stroke	L	12 months
P12	50s	F	Caucasian	Stroke	R	10 months
P14	50s	M	Asian	Stroke	L	5 months

M, male; F, female; L, left; R, right, B, bitemporal.

### Ethics statement

The study was approved by the University Health Network Research Ethics Board, reference number 20-5618 (Toronto, Canada) and registered at ClinicalTrials.gov (NCT05397873A). Data was collected from July 2021 to November 2022. Written informed consent was obtained from all patients.

### Apparatus and measures

During V1, paracentral retinal sensitivity or PRS (average from the 20 points from the 2 central columns from microperimetry C 10-2 68 stimuli program) was assessed using the Macular Integrity Assessment (MAIA) microperimeter (Centervue, Padova, Italy). Fixation stability (FS) was calculated by the MAIA software as a 63% bivariate contour ellipse area (BCEA 63%). A standard LED fixation target consisting of a small red circle of about 0.76° diameter was presented for microperimetry and fixation tests. To account for learning effect, FS was measured two times and the first results were disregarded. Monocular Best Corrected Visual Acuity (BCVA) was obtained for distance with Early Treatment Diabetic Retinopathy Study (ETDRS) charts at 4 m and for near vision with the 100% contrast Colenbrander continuous print chart. Reading speed was measured using the Minnesota Low Vision Reading Test application (MNRead test, University of Minnesota) (20). Contrast sensitivity was obtained binocularly using the Vision Contrast Test System (VCTS) chart at 1 meter on the 1 cycle/degree (cpd) channel of spatial frequency (21). Quality of visual function estimates were obtained from the Veteran’s Affair Low-Vision Visual Functioning Questionnaire 48 (VA-LV-VFQ 48) (22). At 1-week post-treatment, all the baseline tests (V1) were repeated. Retinal sensitivity using microperimetry was collected 1-month post-BT to better assess the stability of the potential improvements over time. The distance of the TRL from the initial PRL, measured in degrees, was calculated using the recorded microperimeter pictures from the tests.

### Treatment

**Figure 1:**
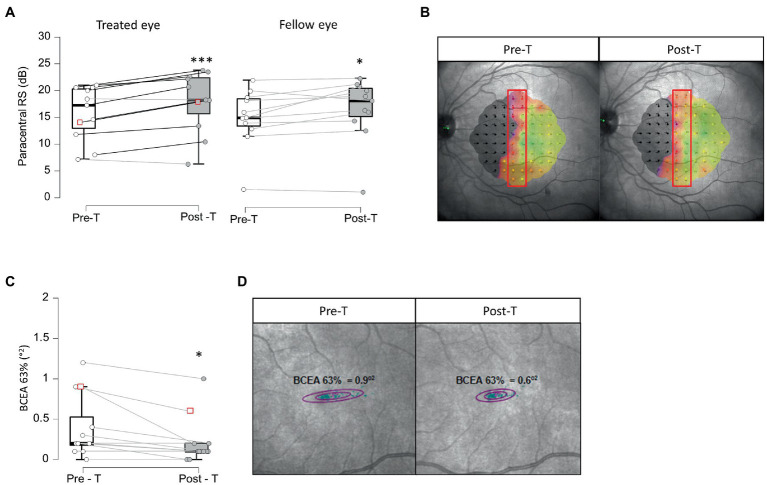
Principle of biofeedback training (BT). **(A)** Pre-BT microperimetry C10-2, left eye. Each green point is an attempt of fixation. Preferred retinal locus (PRL) center is located initially at a 22 dB point, there is splitting of fixation. A trained retinal locus (TRL) was selected toward the seeing retina on a 25 dB retinal point. **(B)** Biofeedback training session as reported by MAIA microperimeter: green dots—fixation attempts. Original PRL (on the left) has more fixation attempts (green dot), while BT training moves fixation points toward the TRL (on the right). **(C)** The center of the new PRL area is located at a 25 dB retinal point. A temporal relocation occurred, and splitting of the fixation was mitigated. The microperimetry shown in C is a 2-year follow up for patient 2.

**Figure 2:**
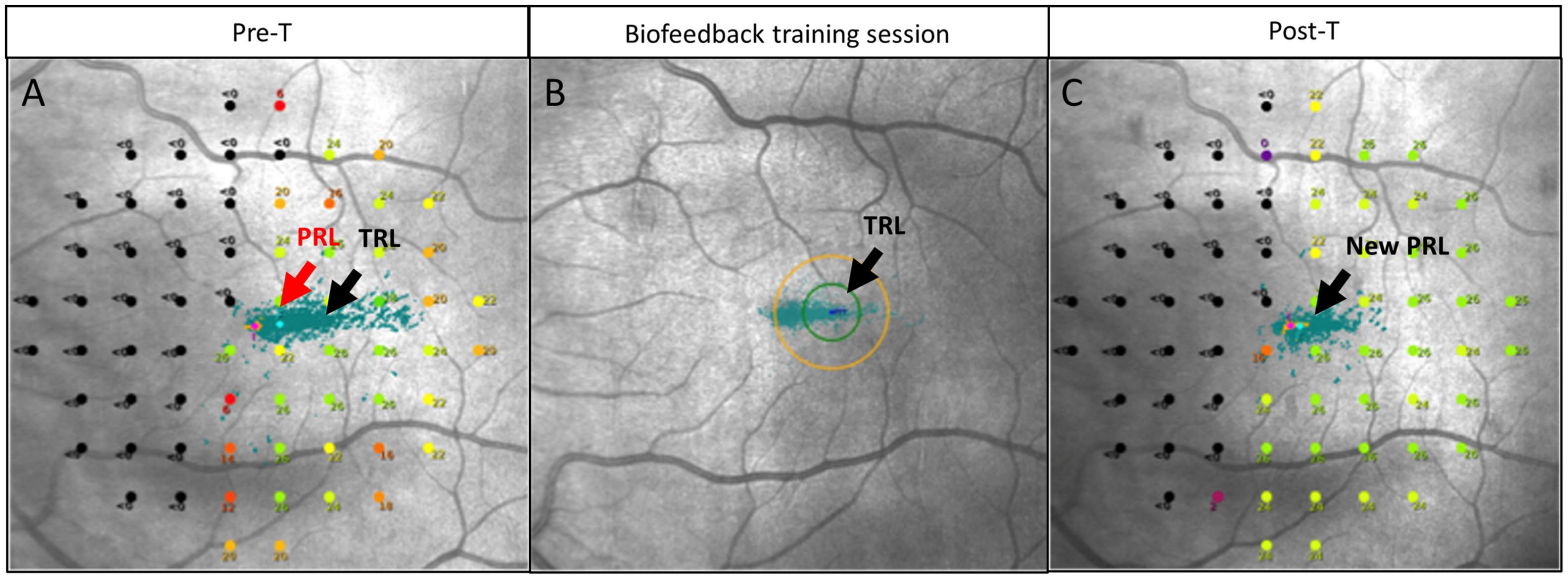
Paracentral retinal sensitivity and fixation stability. Panels **(A,B)** show paracentral retinal sensitivity pre-treatment (Pre-T) and post-treatment (Post-T) in treated and fellow eye in 11 patients **(A)**. Panel **(B)** represents an example of paracentral retinal sensitivity measures within red rectangle, using MAIA microperimeter before (Pre-T) and after (post-T) treatment in one patient, corresponding to red squares in **(A)**. *BF_+0_ > 3; ***BF_+0_ > 10 Panels **(C,D)** show fixation stability measured using the Best Contour Ellipse Area (BCEA) 63% pre- (Pre-T) and post-treatment (Post-T). Panel **(D)** represents an example of BCEA3% measured using MAIA microperimeter before (Pre-T) and after (post-T) treatment in one patient, corresponding to red squares in **(C)**. *BF_-0_ > 3.

BT involved luminous stimulation with auditory feedback performed on the MAIA microperimeter, biofeedback module (Centervue, Padova, Italy; Figure 1). After the patient completed the microperimetry C10-2 test on the same device, the ophthalmologist analyzed the retinal sensitivity map report to determine the retinal trained locus to be used for BT (TRL). This locus should be located no more than 4° from the fovea, and toward the seeing hemifield, to bring the patient’s fixation locus to a larger span area on the retina (Figure 2). The TRL was selected on the screen on top of the microperimetry C10-2 report. The eye ipsilateral to the hemianopsia was trained or, in cases of bitemporal hemianopsia, the eye with better fixation stability.

The BT session involved the presentation of a standard LED fixation point consisting of a small red circle of about 0.76° diameter on the display monitor. The participant was instructed to look at the LED target while listening to the audio feedback. The participant was asked to move the eye toward the TRL under the technician’s scrutiny. The fixation of the patient was monitored in real time on the device’s screen. The auditory feedback changed according to the position of the eye. As the patient was guided to move the eye toward the TRL, the auditory feedback (intermittent beep) would increase frequency progressively, until the TRL was reached, and the auditory feedback would change to a continuous pattern. At this moment, a luminous white dot appeared at the TRL to produce the bimodal stimulation. During this task, the participant would actively control the eye movements and repeat consecutively this fixation in order to exercise the oculomotor control toward and at the TRL.

From V2 to V6, BT was performed weekly for 5 weeks. Each BT session was 20 min long representing a total of 100 min. Pauses were allowed whenever needed.

### Data

Data analysis was based on descriptive statistics including, a measure of central tendency (median) and dispersion (minimum, 25th, 75th percentile and maximum). Eventual missing data were discounted from baseline and outcomes measures. Statistical comparisons between populations were performed by Bayesian paired t-tests (strength of evidence for H1 and Cohen’s d) using JASP software.

## Results

**Figure 3:**
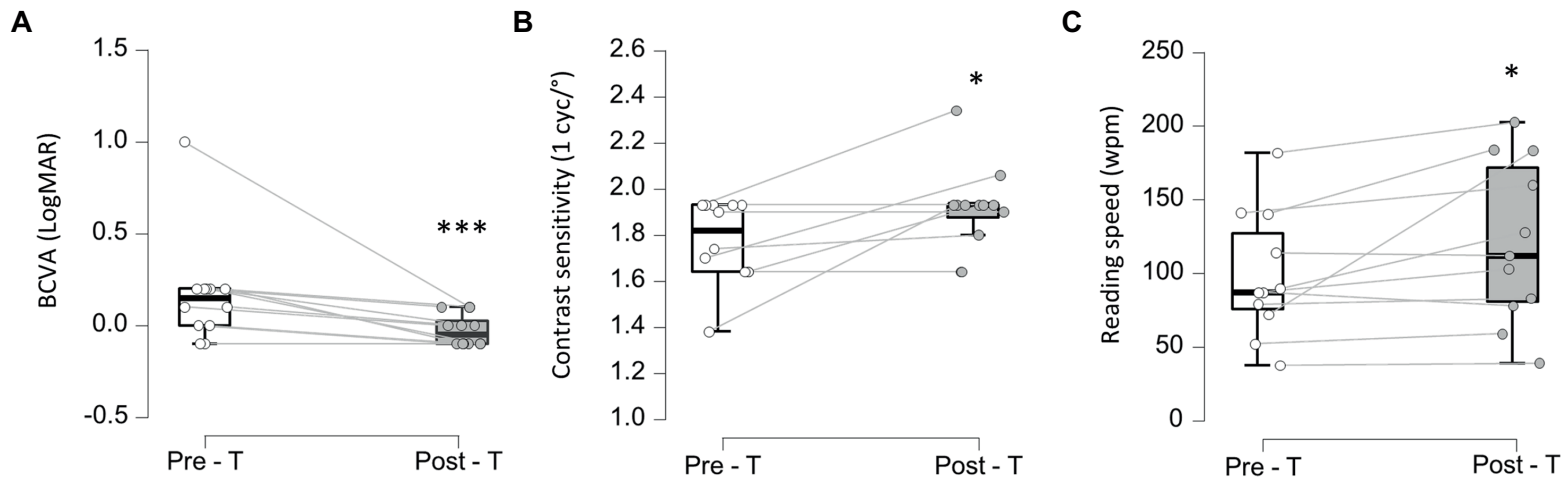
Visual acuity, contrast sensitivity and reding speed. **(A-C)** Pre (Pre-T) and post-treatment (Post-T) measures of visual acuity using Best Corrected Visual Acuity (BCVA) **(A)** ***BF_-0_ > 10, contrast sensitivity at 1cyc/° **(B)** and reading speed (in words per minute—wpm) **(C)** *BF_+0_ > 3.

After a 5-week BT treatment, very strong evidence for an increase in PRS was observed with large effect size in 9/11 patients, from 17.2 ± 5.06 dB [7.15, 12.9, 20.3, 21.0] pre-treatment to 18.3 ± 5.71 dB [6.25, 15.6, 23.4, 23.8, post-T > pre-T BF_+0_ = 65.5, error % = 8.14e-7, d = 1.15, 95%CI (0.37, 2.01)] after treatment in the treated eye, indicating a restoration of visual perception at the border of the blind hemifield (Figures 2A,B; Supplementary Figure 1A). Interestingly, the fellow eye also showed moderate evidence of PRS improvement with medium size effect in 9/11 patients, from 15.1 ± 5.4 dB [1.80, 13.6, 18.6, 22.1] to 18.3 ± 5.91 dB [1.30, 15.3, 20.5, 22.4, post-T > pre-T BF_+0_ = 4.01, error % = 2.10e-4, d = 0.60, 95%CI (0.08, 1.26)] after treatment (Figure 2A; Supplementary Figure 1B). The stability of gaze fixation also improved with moderate evidence and medium size effect as BCEA 63% decreased from 0.20 ± 0.39°^2^ [0.00, 0.17, 0.52, 1.20] at baseline to 0.10 ± 0.29°^2^, [0.00, 0.10, 0.10, 1.00, post-T < pre-T BF-_0_ = 6.79, error % = 6.46e-4, d = 0.67, 95%CI (0.12, 1.32)] after treatment in 8/12 patients (Figures 2C,D; Supplementary Figure 1C). Although no differences were observed in best corrected visual acuity (BCVA) at far distance (ETDRS chart at 4 m – not shown) after treatment, very strong evidence for near distance BCVA (Colenbrander chart) improvement was observed in 10/12 patients, from 0.15 ± 0.29 logMar [−0.1, 0.0, 0.20, 1.00] to −0.05 ± 0.09 logMar [−0.10,-0.10, 0.02, 0.10, post-T < pre-T BF_-0_ = 43.0, error % = 1.54e-5, d = 1.00, 95%CI (0.30, 1.76)] after 5 weeks of BT (Figure 3A; Supplementary Figure 2A). Contrast sensitivity at 1 cycle/° also increased (moderate evidence, medium size effect) from 1.82 ± 0.18 logit [1.38, 1.64, 1.93, 1.93] at baseline to 1.91 ± 0.18 logit [1.64, 1.87, 1.93, 2.34, post-T > pre-T BF_+0_ = 4.05, error % = 1.59e^-4^, d = 0.58, 95%CI (0.08, 1.20)] after BT in 6/12 patients (Figure 3B; Supplementary Figure 2B). Reading speed increased in 10/11 patients (P8 could not be assessed) from 87 ± 42.5 wpm [37.5, 75.5, 127, 181] to 112.0 ± 55.1 wpm [39.0, 80.5, 171, 202, post-T > pre-T BF_+0_ = 3.41, error % = 9.55e-5, d = 0.57, 95%CI (0.07, 1.22)] after treatment (Figure 3C; Supplementary Figure 2C).

**Figure 4:**
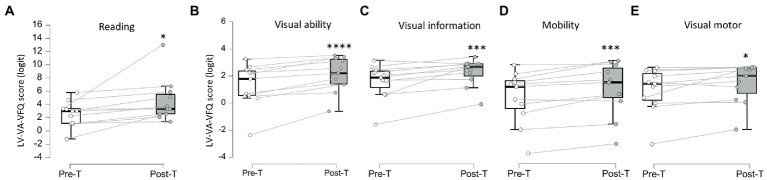
Veteran’s Affair Low-Vision Visual Functioning Questionnaire 48 sub-categories scores. **(A–E)** Pre (Pre-T) and post-treatment (Post-T) measures of subjective self-assessment of reading **(A)**, visual ability **(B)**, visual information **(C)**, mobility **(D)**, and visual motor **(E)**. *BF_+0_ > 3; ***BF_+0_ > 10; ****BF_+0_ > 100.

Overall subjective quality of vision scores (Table 2; Figure 4; Supplementary Figure 3) reported by the patients increased from 1.49 ± 1.91 logit [−3.76, 0.35, 2.43, 5.82] at baseline to 2.51 ± 2.48 logit [−3.04, 1.19, 3.04, 13.0, post-T > pre-T BF_+0_ = 1,289, error % = 5.44e-6, d = 0.65, 95%CI (0.33, 0.98)] in 10/11 patients (P11 could not perform the test). Comparisons for individual sub-categories indicated significant improvement in all sub-sections from the VA-LV-VFQ 48 questionnaire (visual ability, reading, mobility, visual information, and visual motor) when comparing baseline and after treatment, however, a placebo effect cannot be excluded.

**Table 2:** LV-VA-VFQ-48 sub-group scores.

Subsections		Median	Min	Q1	Q3	Max	BF_+0_	Error %	d
Reading	Pre-T	2.99	−1.23	1.13	3.32	5.82	4.61	3.43 × 10^−4^	0.63
	Post-T	3.35	1.41	2.56	5.52	13			
Visual ability	Pre-T	1.78	−2.37	0.5	2.36	3.24	245	1/∞	1.44
	Post-T	2.20	−0.62	1.40	3.21	3.51			
Visual information	Pre-T	2	−1.54	1.25	2.40	3.29	9.15	8.36 × 10^−6^	0.91
	Post-T	2.81	0	2.12	3.04	3.56			
Mobility	Pre-T	1.18	−3.76	−0.44	1.61	2.81	52.1	1.87 × 10^−4^	1.10
	Post-T	1.51	−3.04	0.37	2.55	3.12			
Visuo-motor	Pre-T	1.42	−3.04	0.20	2.19	2.65	4.22	2.56 × 10^−4^	0.61
	Post-T	2.02	−1.96	0.72	2.60	2.65			

The PRL was trained to relocate 2.0 ± 0.4° toward the blind hemifield on the retina. Measures post-treatment indicated a relocation of the PRL 0.14 ± 0.5° within the blind hemifield (Figure 1).

## Discussion

Our study showed significant improvement in paracentral retinal sensitivity, fixation stability, contrast sensitivity, near vision, reading speed, and subjective visual functioning. The treatment consisted of a weekly BT session (20 min) for 5 weeks with audio-luminous stimuli on the MAIA microperimeter. BT was delivered after the critical healing phase post brain-injury (exception for P5 treated 2.5 months post-stroke).

Strong and moderate evidence for increased PRS within the central 4° horizontal and 20° vertical of the new PRL were observed in the treated and fellow eyes, respectively. PRS measured by automated microperimetry (MAIA C10-2 program) is controlled for the loss of fixation, therefore, such improvement of the PRS with medium to large effect size strongly suggests visual field relocation at the border of the blind hemi-field. As the seeing hemi-retina is relocated to the center of the new test, the MAIA would capture the PRS from a better retinal area, representing a better use of the visual functions.

The recovery of visual perception at the border of the scotoma has been observed in individuals with hemianopsia using field restitution rehabilitation approaches, although it requires significantly longer duration of stimulation, typically for several months, representing hundreds of hours of stimulation (23, 24). Other compensatory approaches such as oculomotor training typically takes place over 1-h daily sessions for 1 month, requiring strong commitment (2). Here, visual field relocation, improved fixation stability, improved visual functions, and increased quality of life were observed after 5 weeks, representing a total of 100 min of static luminous stimulation with auditory feedback.

The fast functional visual improvements when compared to the traditional compensatory therapies might be the consequence of the combination of auditory biofeedback and visual stimulation, reinforcing the training effect of new PRL relocation through multisensory processing (25).

Fixation stability significantly improved in accordance with the expected effect of BT which trains the patients to use a new PRL through oculomotor control for activities of daily living (12). Similar improvements of fixation stability are observed in individuals with macular degeneration and PRL relocation through BT (12, 14, 15, 19).

Contrast sensitivity at 1 cycle/° also improved. This could be the consequence of the improvements observed in paracentral fields sensitivity and fixation stability. Such increase in contrast sensitivity was observed in other studies using high-contrast visual stimulation (26, 27).

Parafoveal retinal sensitivity, fixation stability and contrast sensitivity are essential features for proper reading (28). Accordingly, improvement in these three features led to an increase in left-to-right reading speed in 83% (10/12) of our patients with hemianopsia regardless of the side affected (left or right HH). Patients with right hemianopsia (right-sided field loss) have difficulties in shifting their gaze systematically from left to right and show poor sentence tracking whereas patients with left hemianopsia have issues finding the beginning of a new line in right-to-left reading. Our results suggest that such eye movements are improved after BT and hence BT is probably the mechanism which enhances fixation stability and results in better visual functions post treatment.

Overall subjective patient-reported visual function strongly improved, corroborating the results observed with visual function and functional vision outcomes. More specifically, the visual ability and visual mobility subgroups showed the highest effect size, suggesting that the patients show improved navigation and orientation. Consistent with the medium size of improvement in reading speed, the patient-reported score of reading ability also moderately increased after BT.

Our results are unlikely due to a learning/adaptation effect of the visual tests as baseline and after treatment assessments at the clinic were separated by a minimum of 5 weeks, above the learning effect time window shown to last for up to 1 week (29). Audiovisual stimulation with BT efficiently improved oculomotor control toward pre-designated targets. Improved oculomotor control resulted in better fixation stability of the eyes. BT allowed relocation of parafoveal visual fields, enlarging the central vision. Larger paracentral fields, better fixation stability and an increased contrast sensitivity translate into improved reading but also better navigation and orientation, and consequently, increased quality of visual function.

The audiovisual sensory BT is a therapy used in low vision for more than 10 years, showing good results for near and distance vision in individuals with macular degeneration and other low vision conditions (13–19). A limitation of this study was the small number of participants. As a safe and cost-efficient rehabilitation technique and following validation with larger studies, BT could provide a relevant visual rehabilitation for patients with hemianopsia.

## Data availability statement

The raw data supporting the conclusions of this article will be made available by the authors, without undue reservation.

## Ethics statement

The studies involving human participants were reviewed and approved by University Health Network Research Ethics Board. The patients/participants provided their written informed consent to participate in this study.

## Author contributions

MMi is the first author, current clinical fellow seeing the patients and contributing to the manuscript preparation. YP worked for 2 years as a clinical fellow seeing patients for the project, obtaining consents and questionnaires. AS worked as a technician and optometrist assessing the patients and obtaining consents and questionnaires. MMa is an optometrist and occupational therapist and was involved in the design of the study and recruitment of patients. SM is the professor involved in the design of the study and manuscript confection. MR is the co-senior author involved in design, statistical analysis, and manuscript preparation. MD-N idealized the study, was the principal investigator supervising and participating in all activities, including manuscript preparation. All authors contributed to the article and approved the submitted version.

## Funding

This work was supported by UHN Foundation (MR).

## Conflict of interest

The authors declare that the research was conducted in the absence of any commercial or financial relationships that could be construed as a potential conflict of interest.

## Publisher’s note

All claims expressed in this article are solely those of the authors and do not necessarily represent those of their affiliated organizations, or those of the publisher, the editors and the reviewers. Any product that may be evaluated in this article, or claim that may be made by its manufacturer, is not guaranteed or endorsed by the publisher.
