# Supplementary figures and images for: Innovative vision rehabilitation method for hemianopsia: Comparing pre- and post audio-luminous biofeedback training for ocular motility improving visual functions and quality of life
Source: Front Neurol. 2023 Apr 11;14:1151736. doi: 10.3389/fneur.2023.1151736 (PMC10126773; doi:10.3389/fneur.2023.1151736)

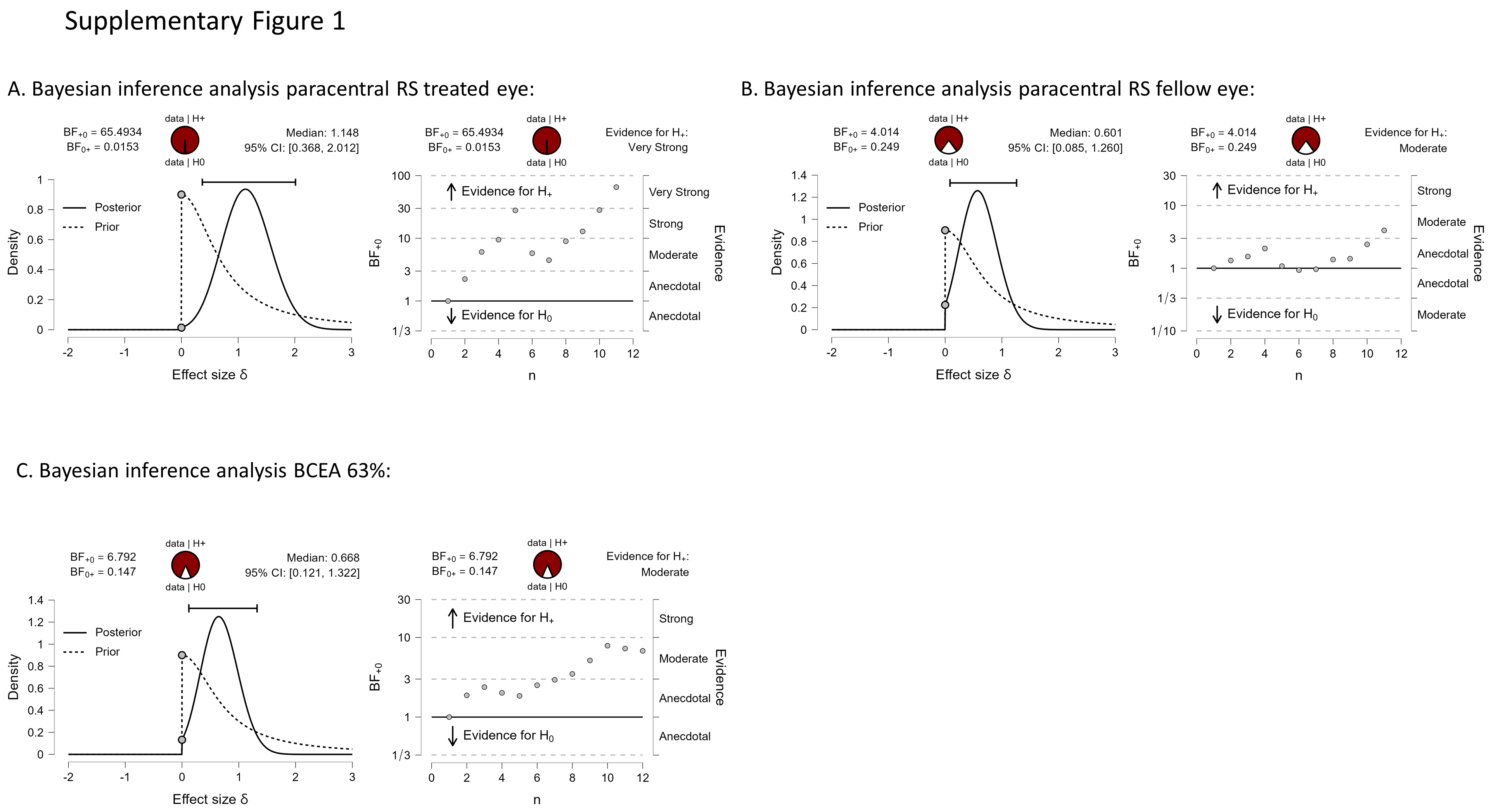

Supplement: Supplementary file 1 [file Image_1.tif]

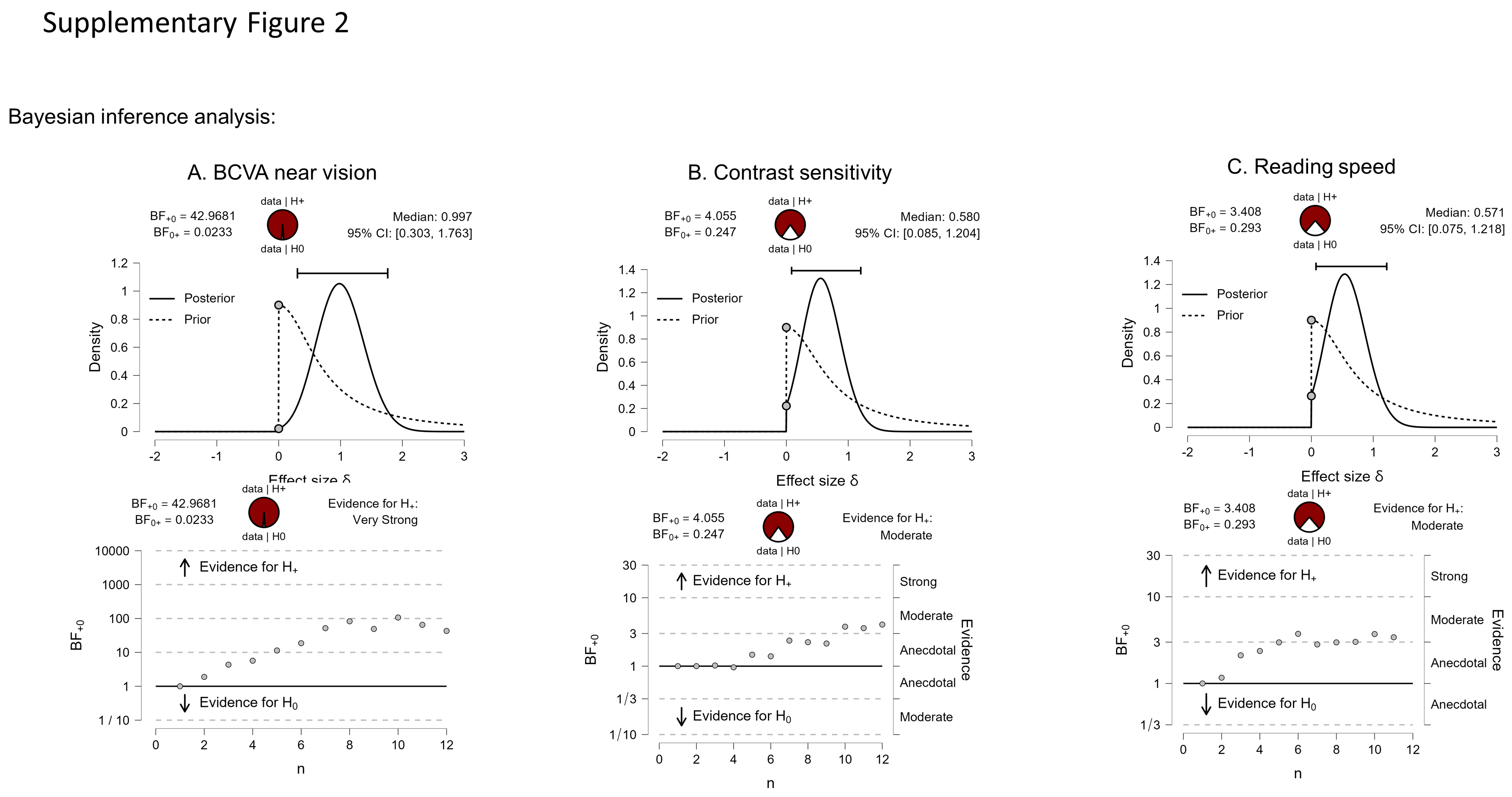

Supplement: Supplementary file 2 [file Image_2.tif]

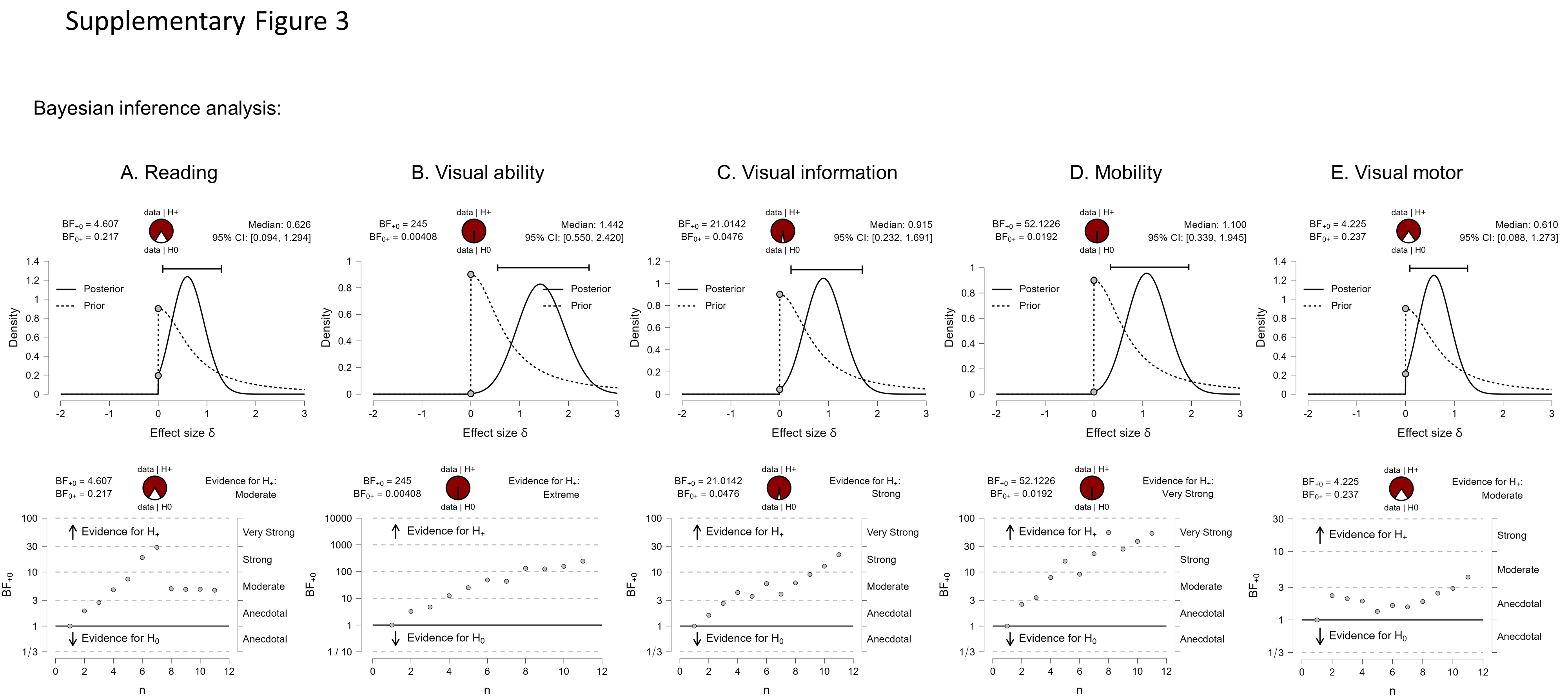

Supplement: Supplementary file 3 [file Image_3.tif]
